# APOBEC3G and APOBEC3F Require an Endogenous Cofactor to Block HIV-1 Replication

**DOI:** 10.1371/journal.ppat.1000095

**Published:** 2008-07-04

**Authors:** Yanxing Han, Xiaojun Wang, Ying Dang, Yong-Hui Zheng

**Affiliations:** Department of Microbiology and Molecular Genetics, Michigan State University, East Lansing, Michigan, United States of America; Aaron Diamond AIDS Research Center, United States of America

## Abstract

APOBEC3G (A3G)/APOBEC3F (A3F) are two members of APOBEC3 cytidine deaminase subfamily. Although they potently inhibit the replication of *vif*-deficient HIV-1, this mechanism is still poorly understood. Initially, A3G/A3F were thought to catalyze C-to-U transitions on the minus-strand viral cDNAs during reverse transcription to disrupt the viral life cycle. Recently, it was found more likely that A3G/A3F directly interrupts viral reverse transcription or integration. In addition, A3G/A3F are both found in the high-molecular-mass complex in immortalized cell lines, where they interact with a number of different cellular proteins. However, there has been no evidence to prove that these interactions are required for A3G/A3F function. Here, we studied A3G/A3F-restricted HIV-1 replication in six different human T cell lines by infecting them with wild-type or *vif*-deficient HIV-1. Interestingly, in a CEM-derived cell line CEM-T4, which expresses high levels of A3G/A3F proteins, the *vif*-deficient virus replicated as equally well as the wild-type virus, suggesting that these endogenous antiretroviral genes lost anti-HIV activities. It was confirmed that these A3G/A3F genes do not contain any mutation and are functionally normal. Consistently, overexpression of exogenous A3G/A3F in CEM-T4 cells still failed to restore their anti-HIV activities. However, this activity could be restored if CEM-T4 cells were fused to 293T cells to form heterokaryons. These results demonstrate that CEM-T4 cells lack a cellular cofactor, which is critical for A3G/A3F anti-HIV activity. We propose that a further study of this novel factor will provide another strategy for a complete understanding of the A3G/A3F antiretroviral mechanism.

## Author Summary

Cytidine deaminases are host enzymes that remove the amino group from the cytidine base on single-stranded DNA or RNA, resulting in a replacement of the cytidine with a uracil. Such replacement may alter the amino acid–coding sequence of the gene and change protein function. It has been well documented that APOBEC1 and AID play very important roles in protein metabolism and immune response via this mechanism. Interestingly, recent advances in retroviral researches have discovered that the seven cytidine deaminases (APOBEC3A to 3H) on human Chromosome 22 can restrict retrovirus replication. In particular, APOBEC3G and APOBEC3F have the most powerful anti–HIV-1 activity and also inhibit other retroviruses, including retrotransposons. They could inhibit viral replication in either a cytidine deamination-dependent or -independent manner, but the precise mechanism remains to be defined. In this report, we found that in a particular human T cell line, APOBEC3G and APOBEC3F failed to block HIV-1 replication. Further analyses indicated that this cell line lacks a cellular factor, which is very critical for APOBEC3G and APOBEC3F antiviral activity. Thus, APOBEC3G and APOBEC3F require a cofactor to inhibit viral replication, and identification of this cofactor will provide an important strategy to decipher this poorly defined antiretroviral mechanism.

## Introduction

Cytidine deaminases are RNA-editing enzymes that target cytosines for conversion to uracils (C-to-U). They belong to the apolipoprotein B mRNA-editing enzyme catalytic polypeptide-like (APOBEC) family, which includes activation-induced deaminase (AID), APOBEC1 (A1), APOBEC2 (A2), a group of APOBEC3 (A3), and APOBEC4 (A4) in humans [1]. A1 is the original member of this family and remains the best characterized. It has the capability to introduce a premature termination codon on apolipoprotein B100 (apoB) mRNA by C-to-U editing to produce a truncated form of this protein [2]. AID is the second member identified. It edits specific “hotspots” on immunoglobulin gene loci in activated B cells to direct somatic hypermutation and isotype class switching to generate different antibodies [3].

The human A3 subgroup contains seven members: A3A, A3B, A3C, A3DE, A3F, A3G, and A3H. All these proteins have antiretroviral activities against different targets, including exogenous retroviruses and endogenous retroelements [1]. The replication of human immunodeficiency virus type 1 (HIV-1) is inhibited by A3B, A3DE, A3F, A3G, and A3H [4]–[11], and A3G shows the most powerful anti-HIV-1 activity [9]. A3G/A3F also blocks various retroelements, including LTR retrotransposons and non-LTR retrotransposons [12]–[18]. Nevertheless, HIV-1 is able to elude this defense mechanism and cause disease in humans for two reasons. First, A3B and A3H are poorly expressed in vivo [5],[7],[19],[20]. Second, HIV-1 produces a viral infectivity factor (Vif) that binds to and mediates the destruction of A3DE, A3F, and A3G in 26S proteasomes via recruitment of the Cullin5 ubiquitin E3 ligase [6], [10], [11], [21]–[25]. Recently, a protein degradation–independent mechanism was also reported [26].

The antiretroviral mechanism of these A3 proteins has been extensively studied. Initially, it was found that A3G proteins deaminate deoxycytidines (dCs) to form deoxyuridines (dUs) on viral minus-strand cDNAs during viral reverse transcription [27]–[30]. These C-to-U mutations could either cause the degradation of the viral minus-strand cDNAs or result in G-to-A hypermutations in the plus-strand viral cDNAs, which create havoc in viral transcripts and produce noninfectious virions. Although this model has been favored for a while, recent investigations suggest that the cytidine deamination may not be absolutely required for A3 antiretroviral activity. First, it was found that anti-HIV activity of A3G/A3F does not correlate with hypermutations, but correlates with the reduction of viral reverse transcripts [31]. In addition, A3F and A3H were shown to inhibit HIV-1 replication in the absence of hypermutations [5],[32]. Second, A3G/A3F and other A3 proteins were also shown to inhibit the replication of some other retroviruses or retrotransposons in the absence of hypermutation [14], [15], [18], [33]–[37]. Further investigations demonstrated that A3G/A3F could interrupt viral reverse transcription by reducing the efficiency of tRNA^lys3^ priming to the viral RNA template, elongation, and DNA strand transfer [38]–[43]. Moreover, they could also block viral integration [42],[44]. However, how these viral enzymatic reactions are inhibited by A3G/A3F is still not clear.

To understand the mechanism of A3G/A3F antiretroviral activity, efforts have made in another direction by isolating their cellular binding proteins, although there is no evidence to prove that they are functionally required. Both A3G and A3F were found in a ∼700 kDa high-molecular-mass (HMM) complex in immortalized cell lines [45],[46]. Unlike A3F, the A3G HMM complexes are RNase-sensitive; when treated with RNase A, they fall apart into 100 kDa low-molecular-mass (LMM) complexes. Biochemical isolations of A3G/A3F-binding proteins from these HMM complexes have generated a list that contains almost 100 different proteins, some of which are shared by A3G and A3F [47]–[50]. However, it is still unclear whether these interactions are essential for A3G/A3F antiviral activities.

In this report, we studied the anti-HIV-1 activity of A3G/A3F in six different human T cell lines. We found that A3G/A3F lost anti–HIV-1 activity in the CEM-derived cell line CEM-T4. Further investigation demonstrated that CEM-T4 cells lacked a cellular cofactor. These results lead to a new direction of investigation on the mechanism of how A3G/A3F inhibits retroviral replication.

## Results

### Identification of an A3G/A3F-Expressing Human T Cell Line Permissive for the HIV-1ΔVif virus

**Figure 1:**
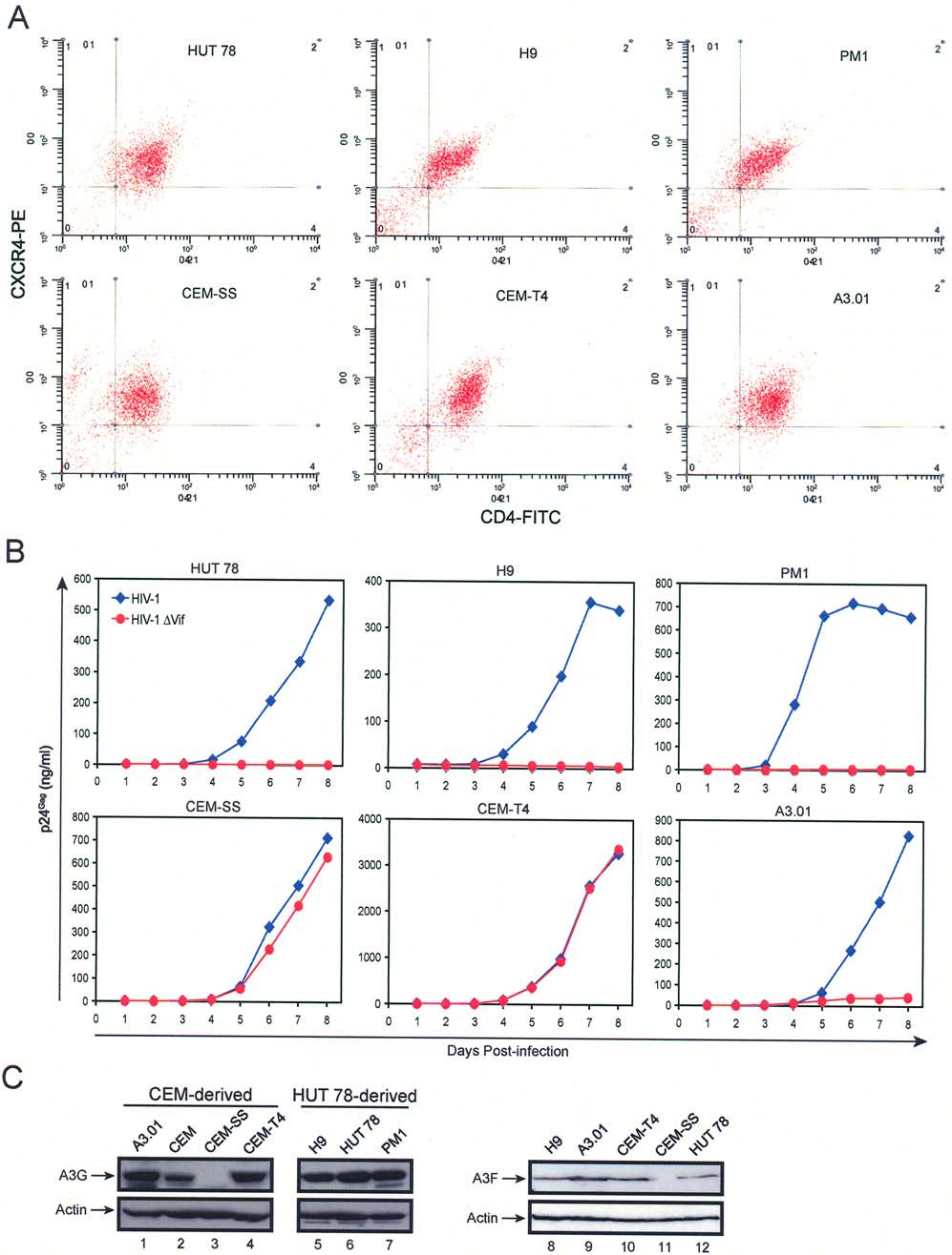
Productive replication of HIV-1ΔVif in CEM-T4 cells. (A) Cell surface expression of HIV-1 receptors. HUT 78, H9, PM1, CEM-SS, CEM-T4, and A3.01 cells were stained with FITC-conjugated anti-CD4 and PE-conjugated anti-CXCR4 antibodies (BD Biosciences). Expression of these surface molecules was determined by flow cytometry. (B) HIV-1 replication. Wild-type or *vif*-defective HIV-1 was produced from 293T cells after transfection with pNL4-3 or pNL4-3ΔVif. HUT 78, H9, PM1, CEM-SS, CEM-T4, and A3.01 cells were then infected with these viruses, and viral production was monitored daily using a p24^Gag^ ELISA for 8 d. Results shown are from 1 of 3 independent experiments. (C) A3G and A3F expression. Cell lysates were prepared from indicated human T cell lines, and the expression of A3G or A3F was determined by Western blotting.

To study A3 protein antiretroviral activity, six human T cell lines, including HUT 78, HUT 78–derived H9 and PM1, and CEM-derived CEM-SS, CEM-T4, and A3.01, were selected for HIV-1 infection. HUT 78 and H9 cells are nonpermissive cells because they express A3G and restrict HIV-1ΔVif virus replication; A3.01 cells are semipermissive cells because the HIV-1ΔVif virus is not completely restricted although it expresses A3G; and CEM-SS cells are permissive cells because they do not express A3G and no longer restrict HIV-1ΔVif replication [51]. CEM-T4 is a natural subclone of CEM and isolated by its relatively high CD4 expression (Paul Clapham, personal communication). The permissiveness of PM1 and CEM-T4 for the HIV-1ΔVif virus has not been determined. To understand whether there is a significant difference in CD4 levels in these cells, the surface expression of CD4 and CXCR4 was determined by flow cytometry. No significant difference in CD4 and CXCR4 expression was observed, although CEM-T4 cells did show a slight high CD4 expression (Figure 1A).

Next, these cells were infected with wild-type or *vif*-defective HIV-1, and viral replication curves were determined for 8 d. Although a robust replication of the wild-type virus was observed in all six cell lines, a significant variation in *vif*-defective virus replication was found (Figure 1B). The replication of HIV-1ΔVif was completely restricted in HUT 78, H9, and PM1 cells; less severely restricted in A3.01 cells; and not restricted at all in CEM-SS and CEM-T4 cells. This result indicated that CEM-T4 should belong to the permissive cell type with no A3G/A3F expression.

To confirm this, A3G and A3F expressions were determined by Western blotting. High levels of A3G were detected in CEM, A3.01, H9, HUT 78, and PM1 cells, and no A3G expression was detected in CEM-SS cells (Figure 1C), which is consistent with previous observations. Strikingly, a high-level of A3G expression was also detected in CEM-T4 cells. In addition, A3F expression was also detected in CEMT4 as well as H9, A3.01, and HUT 78 cells, but not in CEM-SS cells (Figure 1C). These unexpected results caused us to conclude that although CEM-T4 cells express A3G/A3F, they are unable to block HIV-1 replication.

### Characterization of A3G from CEM-T4 Cells

**Figure 2:**
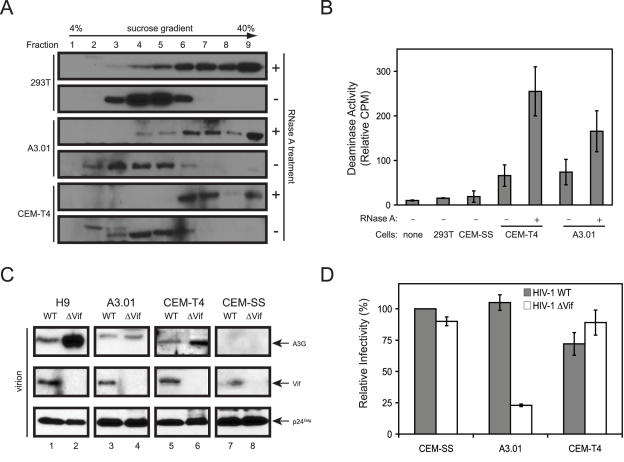
Characterization of A3G in CEM-T4 cells. (A) Isolation of A3G protein complexes. Cytosolic fractions were prepared from CEM-T4, A3.01, and 293T cells transfected with human A3G expression vector. These lysates were treated or untreated with RNase A and then loaded on the top of a 4% to 40% sucrose gradient. After centrifugation at 200,000*g* for 4 h, 9 fractions were collected, and A3G expression was determined by Western blotting. (B) A3G deaminase activity. Cell lysates were prepared from indicated cell lines with or without RNase A treatment and then subjected to scintillation proximity-based cytidine deaminase assay. Error bars represent standard deviations in 3 independent experiments. (C) Production of HIV-1 from human T cell lines. H9, A3.01, CEM-SS, and CEM-T4 cells were infected with wild-type or *vif*-defective HIV-1 carrying a neomycin-resistant gene, and stable cell lines were generated by G418 selection. Viruses were then collected from these cultures, and virion-associated proteins were determined by Western blotting. (D) Viral infectivity assay. Viruses produced from the stable cell lines were collected and used to infect TZM-bI cells. Viral infectivity was determined and normalized to viral input and expressed as relative values to the wild-type virus infectivity from CEM-SS cells. The values for wild-type and *vif*-deficient virus infectivity are 100 and 90 from CEM-SS, 105 and 23 from A3.01 cells, and 72 and 89 from CEM-T4 cells. Error bars represent standard deviations in 3 independent experiments.

Because A3G has much more potent anti–HIV-1 activity than A3F, we decided to further characterize the A3G protein from CEM-T4 cells to understand why A3G could not inhibit HIV-1 replication. First, we cloned and sequenced the *A3G* gene. No mutation was found in this gene from this cell line (unpublished data). Second, we determined whether a defect at a post-translational level was present that could disrupt A3G interaction with other cellular partners. The A3G protein complex was isolated from CEM-T4 cells by sedimentation of cell lysates in sucrose gradients as described previously [46]. A3G was found in the HMM complexes in CEM-T4 cells as it was in 293T and A3.01 cells and, importantly, they were sensitive to conversion to LMM complexes upon RNase A treatment (Figure 2A). This result indicated an intact ability of A3G to interact with cellular RNAs and proteins. Third, we determined A3G cytidine deaminase activity by a scintillation proximity-based assay using an A3G-specific template. As presented in Figure 2B, cells such as 293T and CEM-SS that do not express A3G had marginal deaminase activity; CEM-T4 and A3.01 cells that express A3G had higher levels of activity, which were significantly increased after RNase A treatment. These results confirmed that A3G is present in HMM complexes in these cells (Figure 2A) and further indicated that A3G in CEM-T4 cells is enzymatically active. Thus, no apparent defect on A3G was detected from CEM-T4 cells.

Since the antiretroviral activity of A3G is associated with its presence in virions, we decided to further determine whether A3G could be packaged into HIV-1 virions from CEM-T4 cells. To produce sufficient amounts of virions, cell lines stably producing HIV-1 virions were generated. HIV-1 viruses carrying a neomycin-resistant gene were initially produced from 293T cells after transfection with pNL-Neo or pNL-NeoΔVif. A3.01, H9, CEM-SS, and CEM-T4 cells were then infected with these viruses, and stably infected cell lines were created by G418 selection. Virions were then purified by ultra-centrifugation, and viral proteins were determined by Western blotting. All the cell lines were able to produce viruses as evidenced by the detection of p24^Gag^; Vif was only detectable in samples of the wild-type virus but not the *vif*-deficient virus (Figure 2C). A3G was consistently detected in virions from H9, A3.01, and CEM-T4 cells, but not CEM-SS cells, and more A3G proteins were found in the *vif*-defective than the wild-type virions. The level of A3G encapsidation from CEM-T4 cells was lower than that from H9, but was at least comparable to those from A3.01 cells (Figure 2C; compare lanes 2, 4, 6). Thus, A3G was effectively encapsidated by HIV-1 from CEM-T4 cells. We further compared the infectivity of viruses from CEM-SS, A3.01, and CEM-T4. We found that HIV-1ΔVif infectivity was significantly reduced only in A3.01 cells, not CEM-SS and CEM-T4 cells (Figure 2D). This result indicated that the *vif*-deficient virions produced from CEM-T4 cells were still infectious, which explained why CEM-T4 cells were permissive for HIV-1ΔVif replication.

### Trans-Complementation of CEM-T4 with Other Cellular Factors

**Figure 3:**
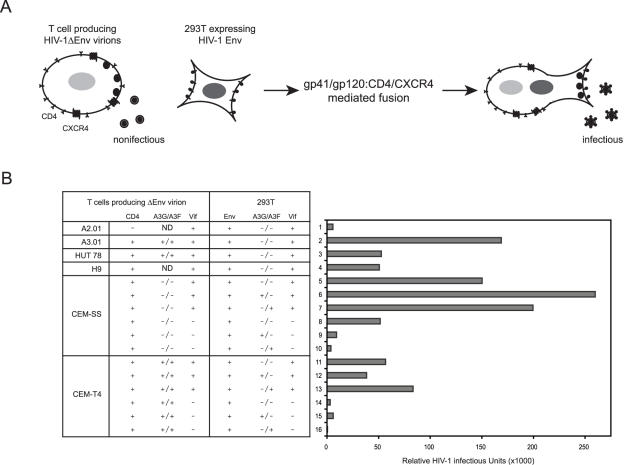
CEM-T4 cells lack an A3G/A3F cofactor. (A) A schematic description for transient trans-complementation assay. T cells were infected with *env*-defective HIV-1 virus pseudotyped with VSV-G, and 293T cells were transfected with the Env-expressing vector pNLΔGag. These two types of cells were cocultured for heterokaryon formation. Infectious particles were then detected by infection of TZM-bI cells. (B) Infectivity of HIV-1 produced from heterokaryons. 293T cells were transfected with either pNLΔGag or pNLΔGagΔVif in the presence or absence of a human A3G or A3F expression vector and then cocultured with A2.01, A3.01, HUT 78, H9, CEM-SS, or CEM-T4 T cells that were infected with VSV-G–pseudotyped *env*-defective HIV-1 expressing or not expressing Vif protein. Infectious particles were collected 48 h later and used to infect TZM-bI cells. Viral infectivity was finally determined by measuring firefly luciferase activity in TZM-bI cell lysates. Results shown here were from 1 of 3 independent experiments.

We developed two hypotheses to explain why CEM-T4 cells are permissive for HIV-1ΔVif replication: 1) CEM-T4 cells lack a cofactor essential for A3G/A3F anti-HIV activity; and 2) CEM-T4 cells express a dominant inhibitor that blocks A3G/A3F activity. A previously described trans-complementation assay was used to test these hypotheses [52],[53]. In this assay, CEM-T4 cells were fused with 293T cells to form heterokaryons. Because 293T can support A3G/A3F antiviral activity, if A3G/A3F antiviral activity is restored in heterokaryons, it would indicate that a cofactor is missing in CEM-T4 cells; otherwise, CEM-T4 cells should express an inhibitor. To ensure that infectious virions are produced exclusively from heterokaryons, we expressed *env*-deficient viral particles (HIV-1ΔEnv) in CEM-T4 cells and HIV-1 Env protein in 293T cells. No infectious particle could be produced from these two cell lines unless they formed heterokaryons by HIV-1 Env and CD4/CXCR4-mediated cell fusion and trans-complemented for the missing viral components (Figure 3A). The infectious viral particles were then detected by infecting the HIV-reporter cell line TZM-bI, which contains an integrated firefly luciferase gene under the control of the HIV LTR.

Initial control experiments were performed with T cells that support A3G/A3F anti–HIV-1 activity. A2.01, a CEM-derived human T cell line that does not express CD4, was used as a negative control, whereas A3.01, HUT 78, and H9 were used as positive controls. Since A2.01 cells should not fuse to 293T cells, no infectious particles should be recovered. Indeed, very low luciferase activity was detected from TZM-bI cells inoculated with culture supernatant from A2.01 and 293T coculture (Figure 3B; lane 1). In sharp contrast, when A2.01 was replaced by A3.01, HUT 78, or H9, 10- to 30-fold higher luciferase activities were detected from TZM-bI, indicating a high efficiency of heterokaryon formation and release of infectious particles from these heterokaryons (Figure 3B; lanes 2–4).

Since CEM-SS cells do not express A3G/A3F, they were further used to test the sensitivity of this system to A3G/A3F and Vif activities. When A3G or A3F was not expressed in 293T cells, infectious particles were recovered from heterokaryons regardless of whether Vif was expressed (Figure 3B; lanes 5 and 8). However, when A3G or A3F was expressed, infectious particles were only recovered from heterokaryons in the presence of Vif (Figure 3B; lanes 6 and 7), not in the absence of Vif (Figure 3B; lanes 9 and 10). These results not only demonstrated the efficiency and accuracy of this trans-complementation assay, but also confirmed the expression of corresponding proteins from different constructs.

Finally, we fused CEM-T4 cells with 293T cells. When Vif was expressed in heterokaryons, infectious particles were recovered regardless of whether A3G or A3F was expressed (Figure 3B; lanes 11–13). In sharp contrast, when Vif was not expressed and A3G or A3F was expressed either from CEM-T4 or 293T, no infectious particles were recovered (Figure 3B; lanes 14–16). These results indicated that A3G/A3F in the heterokaryon between CEM-T4 and 293T could block HIV-1 replication. Thus, we concluded that CEM-T4 cells lack a cellular cofactor required for A3G/A3F anti-HIV activities.

### HIV-1 Replication in CEM-T4 Cells Transiently Expressing Exogenous A3G/A3F

**Figure 4:**
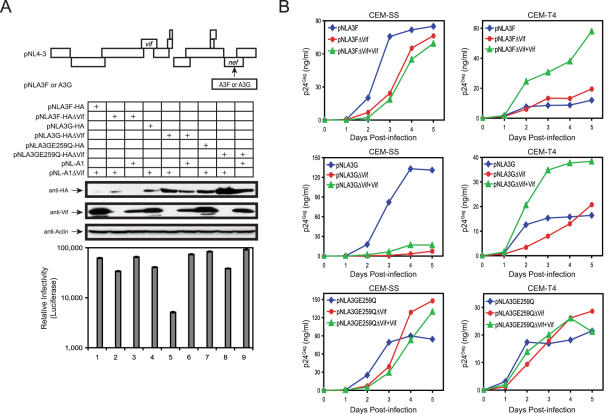
Anti-HIV activity of A3F/A3G proteins expressed *in cis* from an HIV-based vector. (A) Expression of A3G/A3F from an HIV-based vector and the antiviral infectivity in a single round replication assay. An A3F, A3G, or A3GE259Q gene with an HA tag was inserted into the Nef open reading frame in pNL4-3 or pNL4-3ΔVif. These constructs were then cotransfected into 293T cells with either pNL-A1 or its control pNL-A1ΔVif as indicated. Expressions of Vif and A3 proteins in these transfections were determined by Western blotting, and viral infectivity was determined in TZM-bI cells. (B) Replication kinetics of HIV-1 viruses expressing A3G or A3F. CEM-SS and CEM-T4 cells were infected with the same amount of A3G- or A3F-expressing HIV-1 viruses used as in (A); viral growth curves were determined by measuring p24^Gag^ in the supernatants. pNLA3FΔVif + Vif, pNLA3GΔVif + Vif, and pNLA3GE259QΔVif + Vif are viruses produced in the presence of pNL-A1 during transfection of 293T cells.

Although we found that the endogenously expressed A3G/A3F in CEM-T4 cells lost anti-HIV activity, we wanted to know whether this defect is still present when these proteins are overly expressed. We therefore attempted to express A3G or A3F genes transiently in an HIV-based vector. A3F, A3G, or the noncatalytic A3G (A3GE259Q) gene with a 3′ HA tag was inserted into the Nef open reading frame in either pNL4-3 or pNL4-3ΔVif vector so that these A3 proteins could be expressed during HIV-1 replication (Figure 4A; top panel). In total, six different HIV-1 proviral constructs were generated: pNLA3F, pNLA3FΔVif, pNLA3G, pNLA3GΔVif, pNLA3GE259Q, and pNLA3GE259QΔVif.

To test their activities, recombinant viruses were first produced by transfection of 293T cells and viral infectivity was determined. To further confirm A3G/A3F gene function in these vectors, we cotransfected the *vif*-defective version of these vectors with pNL-A1. pNL-A1 is a Vif expression vector created from pNL4-3 with most of the viral genes deleted, including the viral RNA packaging signal, and can only provide Vif expression *in trans* for a single round. High levels of A3G or A3GE259Q expression were detected in transfected 293T cells in the absence of Vif by Western blotting (Figure 4A; middle panels; lanes 5 and 8), and their expressions were decreased by Vif expressed either *in cis* or *in trans* (Figure 4A; lanes 4, 6, 7, 9). The expression of A3F was relatively low (Figure 4A; lane 2), and Vif further decreased this expression (Figure 4A; lanes 1 and 3). Next, recombinant viruses were collected to infect TZM-bI cells for a single-round replication. Overall, these viruses had a very similar infectivity in the presence of Vif (Figure 4A; bottom panel; lanes 1, 3, 4, 6, 7, 9). However, A3 proteins decreased viral infectivity when Vif was absent. The wild-type A3G had the most powerful anti-HIV activity, which reduced viral infectivity by around 10-fold (Figure 4A; lane 5). Both A3F and A3GE259Q mutant reduced viral infectivity by around 2-fold (Figure 4A; lanes 2 and 8). The low anti-HIV activity of A3F could be due to its low expression, and the low activity of A3GE259Q is consistent with previous reports [16], [54]–[56]. Nevertheless, this result confirmed that these constructs expressed functional A3G or A3F proteins.

Next, we infected CEM-SS and CEM-T4 cells with these recombinant HIV viruses. In this experiment, pNLA3 indicates HIV-1 expressing both Vif and A3 proteins, pNLA3ΔVif indicates viruses expressing only A3 protein, and pNLA3ΔVif+Vif indicates viruses expressing Vif provided *in trans* by pNL-A1 and A3 proteins. As presented in Figure 4B, the replication of both pNLA3FΔVif and pNLA3FΔVif+Vif were slightly delayed in CEM-SS cells when compared with pNLA3F, and this result was reversed in CEM-T4 cells; the replication of both pNLA3GE259QΔVif and pNLA3GE259QΔVif+Vif were also slightly delayed in CEM-SS cells during the first 3 d of infection, and all three A3GE259Q-expressing viruses replicated equally well in CEM-T4 cells; the replication of pNLA3GΔVif and pNLA3GΔVif+Vif but not pNLA3G viruses was strictly restricted in CEM-SS cells, and all 3 viruses replicated almost equally well in CEM-T4 cells. The growth curve of these viruses in CEM-SS cells was consistent with their infectivity data in Figure 4A, confirming that A3F only weakly inhibited viral replication due to low expression, A3G potently inhibited viral replication, and A3GE259Q had very low antiviral activity. Nonetheless, since all *vif*-deficient viruses expressing A3G or A3F replicated very well in CEM-T4 cells, these results indicated that A3G/A3F lost their antiviral activity, which further supported that CEM-T4 cells lack a cofactor. The slight delay of pNLA3GΔVif virus replication in CEM-T4 cells could be due to an incorporation of this cofactor from 293T cells that compensated A3G activity during the first round of infection, which provides another piece of evidence that CEM-T4 cells do not express this cofactor.

### HIV-1 Replication in CEM-T4 Cells Stably Expressing Exogenous A3G/A3F

**Figure 5:**
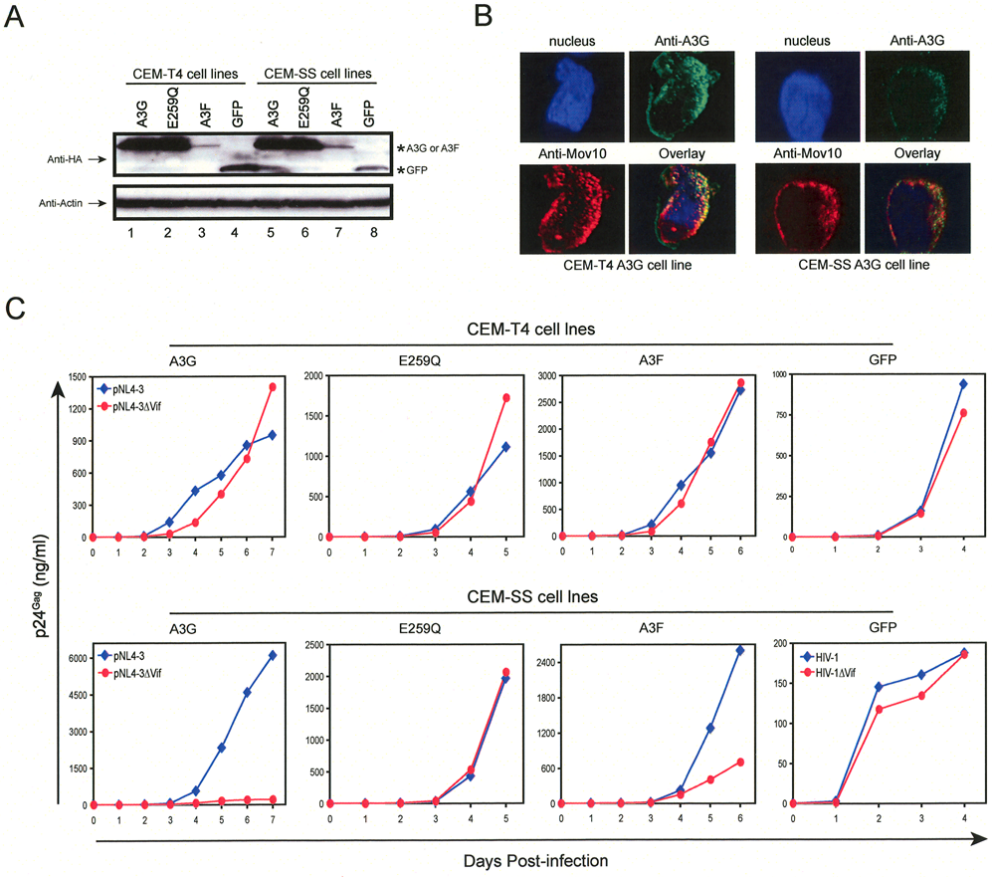
Anti-HIV activity of A3F/A3G proteins expressed *in trans* from a MuLV-based vector. (A) Stable transduction of CEM-T4 and CEM-SS cells by MuLV-based vector. An A3F, A3G, A3GE259Q, or GFP gene with an HA tag was inserted into the pMSCVneo vector, and recombinant MuLV was produced. CEM-T4 and CEM-SS cells were then infected with these viruses, and stably infected cells were selected by G418 treatment. The expression of A3F/A3G proteins in each individual cell line was determined by Western blotting. (B) A3G subcellular localization. CEM-T4 and CEM-SS cells stably transduced with A3G were fixed with formaldehyde and then stained with a mouse anti-A3G monoclonal antibody and a rabbit anti-MOV10 polyclonal antibody. A3G was visualized using Alexa Fluor 488–conjugated secondary antibody (green), and MOV10 was visualized by Alexa Fluor 594–conjugated antibody (red). The cells were also stained with Hoechst 33342 to visualize nuclei (blue). Areas of overlap between A3G and MOV10 appear as yellow. (C) Replication kinetics of HIV-1 in stably transduced cell lines. CEM-T4 and CEM-SS cells stably transduced with A3G, A3GE259Q, A3F, or GFP were infected with the same amount of wild-type or *vif*-deficient HIV-1. Viral growth curves were determined by measuring p24^Gag^ in the supernatants using ELISA.

To further confirm these observations, we stably transduced CEM-T4 or CEM-SS cells with an A3G, A3GE259Q, A3F, or GFP gene by the murine leukemia virus (MuLV)-based vector pMSCVneo. These genes, containing a 3′ HA-tag, were inserted into pMSCVneo, and recombinant MuLV viruses were created by transfection of Phoenix-AMPHO cell line. Viruses were then used to infect CEM-T4 or CEM-SS cells, and 8 stable cell lines were created by G418 selection. All transduced genes were expressed although the expression of A3F and GFP was lower than that of A3G and A3GE259Q (Figure 5A). It is known that human A3G inhibits MuLV replication, but one group reported that this inhibition might not depend on cytidine deamination [57]. Nevertheless, we wanted to make sure that the tranduced gene did not contain any mutation. The exogenous A3G and A3F genes in CEM-T4 cells were cloned and sequenced, and no mutation was found (unpublished data). Thus, these cell lines should express functional exogenous A3G/A3F proteins.

Another possibility that A3G/A3F lost anti-HIV activity is that they are mislocalized in CEM-T4 cells. To exclude this possibility, we compared A3G subcellular localization in CEM-T4 and CEM-SS cells by confocal microscopy. A3G and A3F are both known as cytoplasmic proteins, and can be found in the mRNA processing (P) bodies [58],[59]. A recent report showed that A3F is colocalized with cellular protein MOV10 [48], which is also a P-body protein [60]. When stable CEM-T4 and CEM-SS cells expressing A3G were stained with anti-A3G and anti-MOV10 antibodies, A3G protein was found in the cytoplasm of both cell lines and was colocalized with MOV10 (Figure 5B). Thus, A3G/A3F should not be mislocalized in CEM-T4 cells.

Finally, we determined HIV-1 replication in these cell lines. As presented in Figure 5C, both wild-type and *vif*-deficient HIV-1 replicated equally well in CEM-T4 cells expressing A3G, A3GE259Q, A3F, or GFP, suggesting that the *vif*-deficient virus is not restricted by any of these genes. However, in CEM-SS cells, although the replication of *vif*-deficient virus was not restricted by GFP or A3GE259Q, it was severely restricted by A3G or A3F. The anti-HIV activity of A3F in CEM-SS cells was relatively lower than that of A3G, which could be due to its low expression as shown in Figure 5A. In addition, the poor activity of A3GE259Q further confirmed that this noncatalytic A3G mutant has poor antiviral activity. Thus, we concluded that A3G/A3F failed to inhibit HIV-1 replication in CEM-T4 cells even though they were overexpressed.

## Discussion

In this report, we studied A3G/A3F anti–HIV-1 activity in 6 different human T cell lines. We found that in one cell line, CEM-T4, A3G/A3F lost anti–HIV-1 activity due to the absence of a cellular factor, which is very critical for A3G/A3F anti-HIV activity.

Although A3G/A3F potently inhibits HIV-1 replication, this mechanism is still poorly defined. A3G/A3F has two conserved zinc-binding domains. In the process of blocking HIV-1 replication, these two domains have different functions. The N-terminal domain has a high affinity for RNAs, which normally serves as a virion-packaging signal, and the C-terminal domain has cytidine deamination activity, which is the real catalytic domain [61]–[63]. Initially, it was found that the noncatalytic A3G mutant E259Q still retained intact anti-HIV activity, suggesting that the cytidine deaminase activity is not required for antiviral activity [64]. However, this result could not be reproduced by the other investigators [16], [54]–[56], and we also found that the E259Q mutant had very marginal anti-HIV activity (Figures 4B and 5C). Nevertheless, it is clear that A3G could inhibit the replication of hepatitis B virus and human T cell leukemia virus type 1 in the absence of cytidine deamination [36],[37], and many similar cases have been found in blocking HIV-1, adeno-associated virus, and retrotransposon replications by different A3 proteins [5], [14], [15], [18], [32]–[35]. Thus, although cytidine deamination is not required, the cytidine deaminase activity is absolutely required for A3 antiretroviral activities.

As introduced before, A3G/A3F can reduce the accumulation of HIV-1 cDNAs, which correlates well with their anti-HIV activity. Previously, two groups reported that uracil DNA glycosylases-2 (UNG), a host DNA repair enzyme, is involved in the degradation of viral cDNAs containing uracils [65],[66]. However, another two groups obtained conflicting results and dismissed the role of this enzyme in A3G antiviral activity [16],[67]. In addition, A3G/A3F were shown to inhibit tRNA^lys3^ priming, elongation, or DNA strand transfer during reverse transcription and viral integration [38]–[44]. How A3G/A3G can virtually disrupt these critical reactions in the viral life cycle needs to be understood. Notably, the presence of an A3G/A3F antiretroviral cofactor may help decipher this poorly defined mechanism. One possibility is that this cofactor has nuclease activity that directly degrades viral reverse transcripts. Alternatively, it may increase the affinity of A3G/A3F to viral RNAs or cDNAs so that they can compete with viral reverse transcriptase and integrase for their substrates. Although it was expected that A3G/A3F would specifically interact with viral RNAs or cDNAs to block HIV-1 replication, in vitro study with recombinant A3G proteins failed to demonstrate such specificity [68],[69]. In the case of another deaminase, A1, it has been shown that its editing activity is a highly sequence-specific process dependent upon the primary, secondary, and perhaps even tertiary structure of the RNA substrate. Further investigations have identified two host factors that are required to complement A1 for apoB mRNA editing: ACF (APOBEC1 complementation factor) and its splice variant ASP (APOBEC1 stimulating protein) [70],[71]. ACF is very homologous to the RNA-binding protein GRY-RBP and binds to the U-rich “mooring” sequence of apoB mRNA [71]. Thus, similar cofactors may also be required for A3G/A3F.

Two groups have reported that human A3G/A3F could inhibit yeast LTR retrotransposon Ty1 in *Saccharomyces cerevisiae* [12],[17]. Whether a similar cofactor is required for A3G/A3F antiretroviral activity in yeast therefore becomes an interesting question. If it is required, it would imply that a highly conserved antiviral ortholog gene exists in different organisms. Otherwise, it may indicate that this cofactor is very specific for HIV-1. The latter possibility could also suggest that A3G/A3F uses different antiviral mechanisms to target different retroviruses. Nevertheless, knowledge of the cofactor involved in the process of blocking HIV-1 replication by A3G/A3F is critical to our understanding of HIV pathogenesis. Further characterization of this cofactor will lead to a complete understanding of A3G/A3F anti-HIV activity.

## Materials and Methods

### Plasmids, Cell Lines, and Viruses

HIV-1 proviral constructs pNL4-3 and pNL4-3ΔVif and human A3G or A3F expression pcDNA3.1-V5-6XHis vectors have been described previously [6],[11]. The noncatalytic A3G mutant (A3GE259Q) was created by site-directed mutagenesis in pcDNA3.1 vector. pNL-Neo, pNL-NeoΔVif, pNLA3G, pNLA3GΔVif, pNLA3F, pNLA3FΔVif, pNLA3GE259Q, and pNLA3GE259QΔVif were created by replacing the firefly luciferase gene in pNL-Luc and pNL-LucΔVif with a neomycin-resistant gene or an A3G, A3F, or A3GE259Q gene containing a 3′ HA-tag by NotI/XhoI digestion, respectively. In addition, an A3F, A3G, A3GE259Q, or GFP gene with a 3′ HA tag was inserted into the pMSCVneo vector by EcoRI/XhoI digestion. pNL4-3ΔGag and pNL4-3ΔEnv were created by SphI/AgeI double digestion or NheI single digestion of pNL4-3, followed by large Klenow fragment treatment before T4 ligation. The NheI site is still active in pNL4-3ΔEnv although the *env* gene was inactivated by frame-shift. To create pNL4-3ΔGagΔVif and pNL4-3ΔEnvΔVif, the parental plasmids were digested with PfiMI and filled in with a linker from annealing two DNA oligonucleotides (5′-CTAGCTAGCTAGCCGGCAGA-3′, 5′-GCCGGCTAGCTAGCTAGTCT-3′).

The HIV indicator cell line TZM-bI and human T cell lines HUT 78, H9, PM1, CEM-SS, CEM-T4, A3.01, and A2.01 were from the National Institutes of Health (NIH) AIDS Research and Reference Reagent Program. The Phoenix-AMPHO cell line was from Dr. G. Nolan (Stanford University). T cell lines were cultured in RPMI 1640 with 10% fetal bovine serum (HyClone). Phoenix-AMPHO, 293T, and TZM-bI were cultured in DMEM with 10% bovine calf serum (HyClone).

HIV-1 or MuLV viruses were produced from 293T or Phoenix-AMPHO cells by the standard calcium phosphate transfection.

### HIV-1 Infection of Human T Cell Lines

A total of 1×10^5^ cells were incubated with 100 ng wild-type or Vif-defective HIV viruses at 37°C for 3 h. After removal of the inocula, followed by 3 extensive washings, cells were cultured in 24-well plates for 8 d. Culture supernatants were then collected daily for measurement of p24^Gag^ by ELISA.

### Cytidine Deaminase Assay

A scintillation proximity-based assay was used as described previously [46],[72].

### Western Blotting

The rabbit anti-human A3G polyclonal antibody was from the NIH AIDS Research and Reference Reagent Program. The mouse anti-human A3F polyclonal antibody was from Abnova, Taiwan. Actin was detected by a polyclonal antibody (C-11; Santa Cruz Biotechnology). HIV-1 p24^Gag^ and Vif were detected by antibodies (nos. 3537 and 6459) from the NIH AIDS Research and Reference Reagent Program. HRP-conjugated anti-rabbit or mouse IgG secondary antibodies were from PIERCE. Detection of the HRP-conjugated antibody was performed using Supersignal Wetpico Chemiluminescence Substrate kit (PIERCE).

### Heterokaryon Formation

A previously established protocol was adopted [52],[53]. Briefly, 293T cells were seeded in 6-well plates at 8×10^5^/well in 2 ml medium. After 12 h, cells were transfected with 6 μg of HIV Env expression vector pNL4-3ΔGag or pNL4-3ΔGagΔVif in the presence or absence of A3G or A3F expression vector and washed with PBS 4 h later. Simultaneously, 8×10^5^ T cells were infected with 500 ng of VSV-pseudotyped Env-defective HIV-1 from pNL4-3ΔEnv– or pNL4-3ΔEnvΔVif–transfected 293T cells at 37°C for 3 h. After removal of the inocula and extensive washing, infected T cells were added to the Env-expressing 293T cell culture. After 48 h, supernatants from these cocultures were collected to infect TZM-bI cells. Viral infectivity was finally determined by measuring cellular luciferase activities after another 48 h.

### Immunocytochemistry

CEM-T4 cells stably expressing exogenous A3G from the pMSCVneo vector were fixed in a buffer (5% formaldehyde+2% sucrose in PBS). Fixed samples were permeabilized for 30 min at room temperature in a permeabilization buffer (1% Triton X-100, 10% sucrose in PBS) prior to incubation with antibodies. Cells were then stained with a mouse anti-A3G monoclonal antibody at 1∶100 (ImmunoDiagnostics, obtained from the NIH AIDS Research and Reference Reagent Program) and a rabbit anti-MOV10 polyclonal antibody at 1∶100 (Proteintech Group). Cover slips were washed once in PBS (5 min at room temperature) and incubated with secondary antibodies, including goat anti-mouse IgG Alexa Fluor 488 and goat anti-rabbit IgG Alexa Fluor 594 (Invitrogen). Cells were further stained with 1 μg/ml Hoechst 33342 (Sigma-Aldrich, St. Louis, Missouri, United States of America). Cover slips were then washed twice with PBS and mounted onto microscope slides with glycerol gelatin (Sigma-Aldrich) and were stored at 4°C in the dark until analyzed by a confocal microscope Olympus Fluoview 1000.

### Accession Numbers

The GenBank accession numbers for human APOBEC3G and APOBEC3F are BC024268 and BC038808.
